# EGFR-vIII downregulated H2AZK4/7AC though the PI3K/AKT-HDAC2 axis to regulate cell cycle progression

**DOI:** 10.1186/s40169-020-0260-7

**Published:** 2020-01-28

**Authors:** Hongyu Zhao, Yunfei Wang, Chao Yang, Junhu Zhou, Lin Wang, Kaikai Yi, Yansheng Li, Qixue wang, Jin Shi, Chunsheng Kang, Liang Zeng

**Affiliations:** 1 Department of Neurosurgery, Tongji Hospital, Tongji Medical College, Huazhong University of Science and Technology, Wuhan, Hubei China; 2 Department of Neurosurgery, Tianjin Medical University General Hospital, Laboratory of Neuro-Oncology, Tianjin Neurological Institute, Key Laboratory of Post-trauma Neuro-repair and Regeneration in Central Nervous System, Ministry of Education, Tianjin Key Laboratory of Injuries, Variations and Regeneration of Nervous System, Tianjin, 300052 China; 3 Department of Neurosurgery, Second Affiliated Hospital, Nanchang University, Nanchang, Jiangxi China

**Keywords:** EGFR-vIII, H2AZK4/7AC, Cell cycle, FK228, Glioblastoma

## Abstract

**Background:**

The EGFR-vIII mutation is the most common malignant event in GBM. Epigenetic reprogramming in EGFR-activated GBM has recently been suggested to downregulate the expression of tumour suppressor genes. Histone acetylation is important for chromatin structure and function. However, the role and biological function of H2AZK4/7AC in tumours have not yet been clarified.

**Results:**

In our study, we found that EGFR-vIII negatively regulated H2AZK4/7AC expression though the PI3K/AKT-HDAC2 axis. Because HDAC1 and HDAC2 are highly homologous enzymes that usually form multi-protein complexes for transcriptional regulation and epigenetic landscaping, we simultaneously knocked out HDAC1 and HDAC2 and found that H2AZK4/7AC and H3K27AC were upregulated, which partially released EGFR-vIII-mediated inhibition of USP11, negative regulator of cell cycle. In addition, we demonstrated in vitro and in vivo that FK228 induced G1/S transition arrest in GBM with EGFR-vIII mutation. FK228 could enhance anti-tumour activity by upregulating expression of the tumour suppressor USP11 in GBM cells.

**Conclusions:**

EGFR-vIII mutation downregulates H2AZK4/7AC and H3K27AC, inhibiting USP11 expression though the PI3K/AKT-HDAC1/2 axis. FK228 is an effective and promising treatment for GBM with EGFR-vIII mutation.

## Background

Histone modifications are closely related to the regulation of gene expression and genome function by changing the global chromatin structure. Among these various modifications, histone acetylation is an important component of chromatin decondensation, which greatly influences chromatin structure and function. This acetylation process disturbs histone/DNA interactions and changes histone/histone interactions in the nucleosome, which is relatively stable and plays an important role in regulating gene transcription by providing binding sites for the recruitment of transcription factors [1]. In contrast, histone deacetylation changes the electrostatic properties of chromatin and tends to gene suppression [2]. The acetylation and deacetylation of histones are dynamically regulated by histone acetyltransferases (HATs) or histone deacetylases (HDACs) [3, 4]. Abnormal histone acetylation or deacetylation is closely related to a variety of tumours: acute myeloid leukaemia [5], T cell lymphoma [6], acute promyelocytic leukaemia [7], B-cell lymphomas [8], ovarian carcinoma [9], gastric cancer [10], colorectal tumours [11], gliomas [12], prostate cancer [13], lung cancer [14], breast carcinoma and medulloblastoma [15], oesophageal squamous cell carcinomas [16], and pancreatic cancer [17]. H2AZ, the most conserved variant of H2A, is associated with chromatin integrity and transcriptional regulation [18, 19]. In the yeast *Saccharomyces cerevisiae*, acetylated H2AZ is enriched in the promoter regions of active genes [20]. The role and biological function of H2AZK4/7AC in tumours have not yet been clarified. The results of our study, provide deep insight into the role of H2AZK4/7AC in glioma with EGFR variant III (EGFR-vIII) overexpression.

Glioblastoma (GBM), the most common brain tumors in adults, is one of the most lethal tumours and has a poor prognosis. The traditional treatment for GBM is surgical excision followed by concurrent chemoradiotherapy and adjuvant chemotherapy. However, the benefits of this treatment have been limited. EGFR-vIII, the result of EGF receptor mutation, is mostly involved in GBM; EGFR-vIII occurs in the classic subtype of GBM and is found in approximately one-third of GBMs [21]. The EGFR-vIII mutation was found to continuously activate downstream pathways to promote proliferation, survival, invasion, and angiogenesis [22]. The PI3K-AKT pathway, one of the main downstream signalling pathways of EGFR, is continuously activated when EGFR is mutated [12]. Previous researchers found that PI3K-AKT pathway activation could promote HDAC2 transcription and translation, accelerating the progression of hepatocellular carcinoma [23]. In our study, we found that HDAC2 expression was upregulated in EGFR-vIII-expressing cells and that HDAC2 may be a target for EGFR-activated GBM.

Epigenetic reprogramming in EGFR-activated GBM has recently been suggested to downregulate the expression of tumour suppressor genes [24]. In this study, we confirmed that EGFR-vIII epigenetically silenced ubiquitin-specific protease 11 (USP11) in vitro and in vivo, which mediated tumour suppression though blocking cell cycle progression [25]. EGFR-vIII activated the PI3K-AKT pathway to upregulate HDAC2 expression, which then reduced H2AZK4/7AC level to silence USP11. Recent studies showed that the recruitment of HDAC1 and HDAC2 to chromatin in complexes repressed transcription [26–28]. Therefore, FK228 might be a novel therapeutic option to target EGFR-vIII GBM.

## Methods

### Glioma samples datasets and bioinformatics analysis

Two large glioma cohorts with gene expression profiles and the corresponding clinical information from the Chinese Glioma Genome Atlas (CGGA, n = 301) and the Cancer Genome Atlas (TCGA, n = 702) were enrolled in our study. The cohort whose data was derived from CGGA comprised 23 classical subtype cases, 111 mesenchymal subtype cases, 81 neural subtype cases, 86 proneural subtype cases, 122 grade II cases, 51 grade III cases, and 128 GBM cases. We used data from 690 glioma samples from TCGA, collected from 256 grade II cases, 269 grade III cases and 165 GBM cases. The UCSC was used to determine H2AZ-, H3K27AC-, H3K4me3- binding sites in the human genome.

### Cell culture and lentivirus transfection

The U87 and U251 human GBM cell lines were obtained from the American Type Culture Collection (ATCC, Manassas, VA, USA). N9 primary glioblastoma cells were kindly provided by Professor Xiaolong Fan (Beijing Key Laboratory of Gene Resource and Molecular Development, Laboratory of Neuroscience and Brain Development, Beijing Normal University). U87, U251 and N9 cells encoding mutant EGFR (U87-vIII, U251-vIII and N9-VIII cells, respectively) were produced through the stable transfection of EGFR-vIII lentivirus (GeneChem, Shanghai, China) and selected with puromycin for at least 7 days before use. U87 and U251 cells were cultured in Dulbecco’s modified Eagle medium (DMEM) with 10% foetal bovine serum (FBS, HyClone), while N9 cells were cultured in DMEM/Nutrient mixture F-12 Ham (DMEM:F-12, 1:1 mixture) containing 10% FBS (HyClone). All cells were cultured at 37 °C in 5% CO_2_.

### Transient transfection

SiRNA against HDAC1 or HDAC2 was transfected into cells for 48 h when cells had reached at approximately 70% confluence. Lipofectamine 3000 (Invitrogen) was used according to the manufacturer’s instructions. siRNAs with the following sequences, which were reported in a previous study, were used: siRNA-HDAC1, 5′-CCC GGAGGA AAG UCU GUU A-3′; and siRNA-HDAC2, 5′-CCC AUA ACU UGC UGU UAA A-3′ [29]. We used scrambled siRNA as a control.

### RNA extraction and real-time quantitative PCR (RT-qPCR)

Total RNA was extracted using TRIzol reagent (Life Technologies). Then, cDNA was synthesized using the GoScriptTM reverse transcription system (Promega, USA). Amplification was performed using a QuantStudio™ 3 real-time PCR system (Thermo Fisher Scientific, USA) according to the manufacturer’s instructions. Relative gene expression levels were analysed by the 2^∆∆−Ct^ method [30]. The following primers were used: HDAC1-F: CACATCAGTCCTTCCAATA, HDAC1-R: GCAGCATTCTAAGGTTCT; HDAC2-F: CACCTCCGATTCCGAGCTTT, HDAC2-R: TCCAATATCACCGTCGTAGTAGT; USP11-F: TGTAGAAGAGAACGGACGGC, USP11-R: TCTCCACAAGGAACCAGCTT; and GAPDH-F: GGTGGTCTCCTCTGACTTCAACA, GAPDH-R: GTTGCTGTAGCCAAATTCGTTGT.

### Chromatin immunoprecipitation (ChIP) and ChIP-qPCR assays

ChIP assays were performed using a Millipore ChIP kit (Magna ChIP™ A/G kit, catalogue # 17-10085) according to the manufacturer’s instructions. Anti-H2AZK4/7AC (Cell Signaling Technology, USA), anti-H3K27AC (Cell Signaling Technology, USA) and anti-H3K4me3 (Cell Signaling Technology, USA) antibodies were used for ChIP assays. The following primers for USP11 were used for ChIP-qPCR: Primer 1-F: TTTGATTCTGGCGGAAGCCT, Primer 1-R: AGATGCAACTCGGCGAGAAA; Primer 2-F: TAATGCAACTTTTGGGGGCG, Primer 2-R: GGCGCGTCATAAACTTTGCT; Primer 3-F: GGGCGGACAGCTAGTTTAGTT, Primer 3-R: AGCCTGATCAGAATGCCCTT; Primer 4-F: TCGCAACGTCTGGAAAAGGG, Primer 4-R: CTCCAGGACCGAAACTGGTC; Primer 5-F: AATATGGCAGTAGCCCCGC, Primer 5-R: CCACTTCCGGATTCTGGTCC; and Primer 6-F: AACGAGGCGAGCTTTGTGA, Primer 6-R: GAAGGCTTCCGCCAGAATCA.

### Western blotting

Western blotting was carried out as described previously [31]. Anti-EGFRvIII, anti-P-AKT, anti-H3K27AC, anti-H2AZK4/7AC, anti-H3K4me3, anti-H3, anti-H2AZ (1:1000, Cell Signaling Technology), anti-HDAC1, anti-HDAC2, anti-USP11, anti-GAPDH, anti-P21 (1:1000, Proteintech), anti-CDK6 (1:1000, ABclonal), anti-CDK4 (1:500, AbSci) and anti-Cyclin D1 (1:500, Absin) primary antibodies were used.

### Co- immunoprecipitation (Co-IP) assays

Cells were resuspended in 1 ml of ice-cold RIPA buffer and incubated at 4 °C for 30 min after washing with cold PBS and collection and then centrifuged at 14,000 r/min for 15 min to remove the precipitate. The lysate was precleared by the addition of 1.0 µg of the appropriate control IgG with a 20 µl volume of resuspended protein A/G agarose (Santa Cruz Biotechnology, Protein A/G PLUS-Agarose; sc-2003). The lysate and beads were incubated at 4 °C for 30 min. After centrifugation at 2500 r/min for 5 min at 4 °C, the cell lysates were collected and mixed with rabbit IgG or primary antibodies together with a 20 µl volume of resuspended protein A/G agarose overnight at 4 °C. Then, the beads were washed using RIPA buffer and collected. After boiling, the samples were assessed by Western blotting.

### Flow cytometry assays

After FK228 treatment for 24 h, cells were harvested and fixed in 70% ethanol at 4 °C for 12 h. Then, the cells were transferred to − 20 ℃. Before measurement, cells were centrifuged at 1500 r/min for 5 min to remove the ethanol and then washed twice with D-PBS. Cell debris was collected by centrifugation at 1500 r/min for 5 min. Each sample was incubated at room temperature for 30 min with 300 µl of a 0.05 mg/µl propidium iodide (PI) dye solution.

### Immunofluorescence assays and confocal imaging

The immunofluorescence assay was carried out as described previously [32].

### Nude mouse intracranial model development

Approximately 4-week-old BALB/cA nude mice were purchased from the Animal Center at the Cancer Institute at the Chinese Academy of Medical Science (Beijing, China) and used to establish an intracranial glioma model. A total of 4 × 10^5^ U87 cells transfected with negative control or U87-vIII cells were implanted stereotactically into each mouse using cranial guide screws [33]. FK228 (1 μg/g) in 1% DMSO in PBS was intraperitoneally injected into each mouse in the treatment group every 3 days. The control group received an equal amount of 1% DMSO in PBS through intraperitoneal injection as previously reported [34]. Tumour size was monitored using the IVIS Lumina imaging system (Xenogen, USA) every 7 days. After sacrifice, the brains were carefully removed and immersed in 10% formalin for 24 h. Then, haematoxylin-eosin (H&E) and immunohistochemical (IHC) staining were performed.

### H&E and IHC staining

H&E and IHC staining was performed as previously described [12]. The following primary antibodies were used: anti-EGFR-vIII (1:200, Cell Signaling Technology), anti-P-AKT (1:100, Cell Signaling Technology), anti-H3K27AC (1:50, Cell Signaling Technology), anti-H2AZK4/7AC (1:50, Cell Signaling Technology), anti-HDAC1 (1:100, Proteintech), anti-HDAC2 (1:100, Proteintech), anti-USP11 (1:50, Proteintech), anti-P21 (1:400, Proteintech), anti-CDK6 (1:100, ABclonal), and anti-CDK4 (1:100, AbSci).

### Statistical analyses

GraphPad Prism 7.0 was used to analyse the statistical significance of differences. A two-tailed *t*-test was performed to compare different groups. Kaplan–Meier plots were used for survival analysis, and the log-rank test was performed to test differences in survival. The two-sided Pearson correlation was used for correlation analysis. Heat maps were produced using Cluster 3.0 and Tree View. Experimental data are presented as the means ± SEMs or means ± SDs, and experiments were performed in triplicate. P < 0.05 indicated a statistically significant difference.

## Results

### The EGFR pathway negatively regulated H2AZK4/7AC expression

**Fig. 1:**
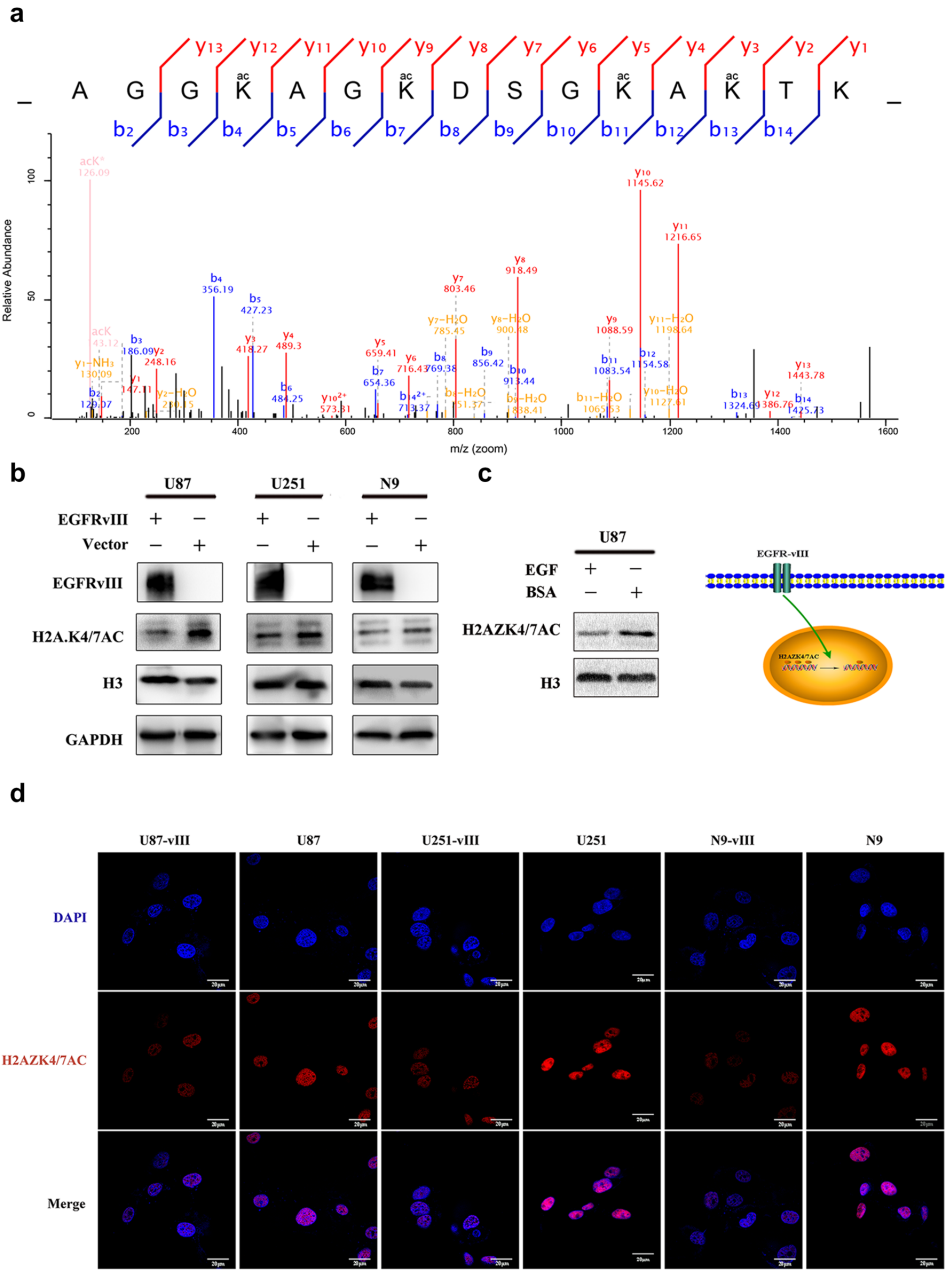
EGFR-vIII negatively regulated H2AZK4/7AC expression. **a** Mass spectrometric analysis revealed that U87-vIII cells presented lower levels of H2AZ acetylation at positions 4 and 7 than U87 cells. **b** Western blotting showed decreased H2AZK4/7AC expression in U87, U251, and N9 cells when EGFR-vIII was overexpressed. **c** U87 cells were stimulated with EGF, following which H2AZK4/7AC was downregulated. **d** Immunoluorescence showed decreased H2AZK4/7AC expression in U87, U251, and N9 cells when EGFR-vIII was overexpressed

Mass spectrometric analysis revealed that the lysine residues of H2AZK4/7AC at positions 4, 7, 11 and 13 in U87 and U87-vIII cells were acetylated. U87-vIII cells presented lower levels of H2AZ acetylation at positions 4 and 7 than U87 cells (Fig. 1a). To determine whether H2AZK4/7AC is negatively regulated by EGFR-vIII, we performed Western blot and immunofluorescence assays. The results showed that the protein level of H2AZK4/7AC was lower in U87, U251, and N9 cells when EGFR-vIII was overexpressed (Fig. 1b, d). Wild-type EGFR (EGFRwt) and EGFR-vIII have similar downstream signalling molecules, and stimulating EGFRwt caused the same downstream changes observed after EGFR-vIII stimulation [35]. To investigate whether H2AZK4/7AC can be downregulated by EGFRwt stimulation, like EGFR-vIII stimulation, EGF was used to stimulate U87 cells, following which H2AZK4/7AC was downregulated (Fig. 1c). EGFR-vIII is important for promoting tumorigenesis and progression, so we hypothesize that H2AZK4/7AC downregulation is associated with malignant GBM progression.

### HDAC2 bound H2AZ and regulated the deacetylation of H2AZK4/7AC

**Fig. 2:**
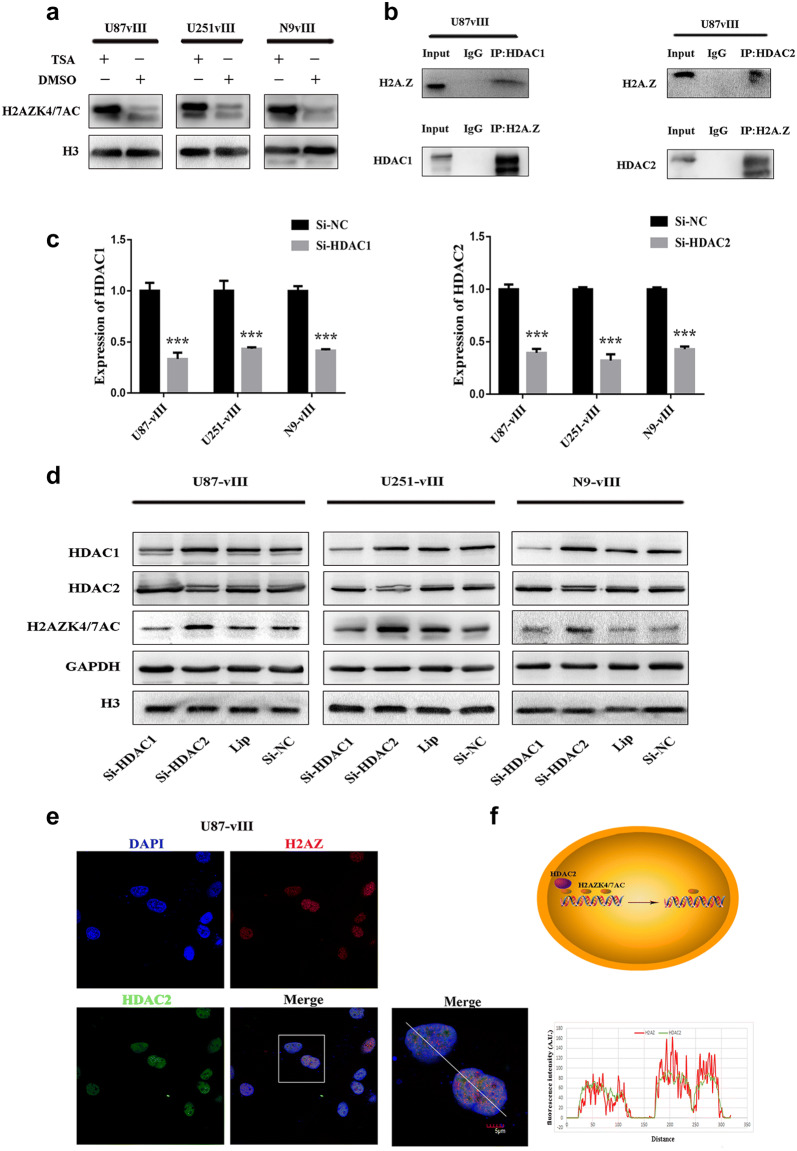
HDAC2 specifically bound H2AZ and regulated the deacetylation of H2AZK4/7AC. **a** U87-vIII, U251-vIII, and N9-vIII cells were treated with TSA (0.2 μm), following which H2AZK4/7AC was significantly upregulated. **b** The endogenous interaction between H2AZ and HDAC1/2 in U87-vIII cells. **c** RT-qPCR showed the efficiency of HDAC1 and HDAC2 siRNA knockout. **d** HDAC2 knockout upregulated H2AZK4/7AC but not HDAC1. **e** Immunofluorescence co-staining and plot profile of HDAC2 and H2AZ expression in U87-vIII cells showed their co-localization in the nucleus. **f** The mechanism by which HDAC2 may downregulate H2AZK4/7AC

First, we used trichostatin A (TSA), a specific HDAC class I/II inhibitor, to treat U87-vIII, U251-vIII, and N9-vIII cells and found that H2AZK4/7AC was significantly upregulated (Fig. 2a). This suggested that HDACs play an important role in regulating H2AZK4/7AC. HDAC1 and HDAC2, which are class I HDACs, exhibit a wide range of expression in tumours and strong enzymatic activity for many histone substrates. To determine whether HDAC1 and/or HDAC2 can specifically regulate H2AZK4/7AC levels, we first performed Co-IP assays and found an endogenous interaction between H2AZ and HDAC1/2 in U87-vIII cells (Fig. 2b). Then, HDAC2 knockdown, the effect of which was verified by using RT-qPCR, in U87-vIII, U251-vIII and N9-vIII cells (Fig. 2c) up-modulated H2AZK4/7AC in U87-vIII, U251-vIII and N9-vIII cells (Fig. 2d). Although HDAC1 can bind H2AZ, HDAC1 and HDAC2 do not have the same enzyme activity against H2AZK4/7AC (Fig. 2d). Immunofluorescence co-staining and a plot profile of HDAC2 and H2AZ expression in U87-vIII cells also showed their co-localization in the nucleus (Fig. 2e). Generally, these data showed that HDAC2 can specifically downregulate H2AZK4/7AC at the protein level in GBM (Fig. 2f).

### Activation of the EGFR-vIII signal transduction pathway upregulated HDAC2 expression through the PI3K/AKT pathway

**Fig. 3:**
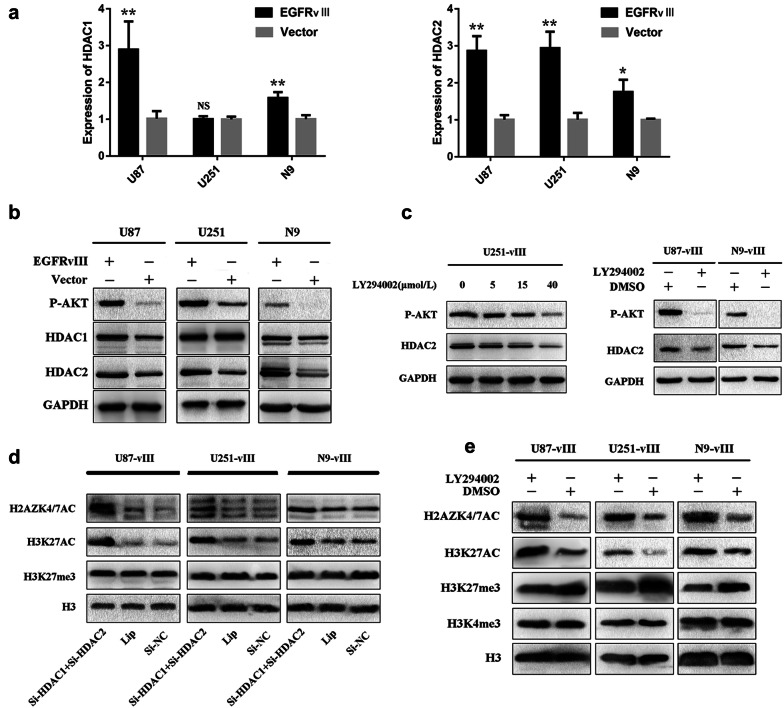
EGFR-vIII upregulated HDAC2 expression through the PI3K/AKT pathway. **a** EGFR-vIII upregulated HDAC2 mRNA levels in U87, U251, N9 cells and HDAC1 mRNA levels in U87 and N9 cells. **b** HDAC2 expression was upregulated at the protein level in U87, U251, and N9 cells, and HDAC1 translation was increased in U87 and N9 cells when EGFR-vIII was activated. **c** HDAC2 expression was decreased in LY294002-treated U87-vIII, U251-vIII, and N9-vIII cells. **d** Simultaneous HDAC1 and HDAC2 knockout upregulated H2AZK4/7AC and H3K27AC expression. **e** PI3K/AKT pathway blocked removed EGFR-vIII-mediated inhibition of H2AZK4/7AC

Class I HDACs are highly expressed in a variety of human tumours [36–40] and play an important role in the malignant progression of cancer. The EGFR signal transduction network is highly complex and consists of multiple signalling pathways. Activation of EGFR in cancer cells can activate several linear pathways, and the PI3K/AKT axis, one of the major downstream pathways, participates in regulating tumour cell proliferation, growth, survival and angiogenesis. EGFR-vIII can continuously activate the PI3K/AKT pathway without EGF stimulation [12]. Activation of the PI3K-AKT pathway upregulated HDAC2 expression at the mRNA and protein levels in hepatocellular carcinoma [23]. To determine whether EGFR-vIII can upregulate the expression of HDAC2 by continuous PI3K/AKT pathway activation, RT-qPCR was performed. The results showed that EGFR-vIII could upregulate the expression of HDAC2 at the mRNA level in U87, U251, and N9 cells (Fig. 3a). The activation of EGFR-vIII also upregulated HDAC1 transcription in U87 and N9 cells but not in U251 cells (Fig. 3a). Western blotting showed that HDAC2 expression was upregulated at the protein level in U87, U251, and N9 cells and that HDAC1 translation was increased in U87 and N9 cells upon EGFR-vIII activation (Fig. 3b). This finding was consistent with the RT-qPCR results. Then, we treated U251-vIII cells with LY294002, a PI3K inhibitor, which induced a dose-dependent increase in HDAC2 expression (Fig. 3c). To investigate whether PI3K/AKT pathway inhibition would have the same effect on HDAC2 in U87-vIII and N9-vIII cells, LY294002 (40 μm) was administered. Inhibition of the PI3K/AKT pathway inhibited HDAC2 expression in U87-vIII and N9-vIII cells (Fig. 3c). These results indicated that EGFR-vIII upregulates HDAC2 transcription and translation through activating the PI3K/AKT pathway.

### Simultaneous knockout of HDAC1 and HDAC2 upregulated H2AZK4/7AC and H3K27AC

HDAC1 and HDAC2 are highly homologous enzymes and usually form multi-protein complexes together with transcription factors and co-repressors for transcriptional regulation and epigenetic landscaping. The HDAC1–HDAC2 dimer has been reported in the CoREST, NuRD and Sin3 complexes, and HDAC1 and HDAC2 may be complementary in terms of their functions in regulating cell proliferation, apoptosis, and differentiation [41]. Deletion of both HDAC1 and HDAC2 leads to severe phenotypes. HDAC1 and HDAC2 have been known to deacetylate histone H3K27 [42, 43]. We inhibited both HDAC1 and HDAC2 and found that H2AZK4/7AC and H3K27AC were upregulated in U87-vIII, U251-vIII and N9-vIII cells (Fig. 3d), while there was no change in H3K27me3 levels (Fig. 3d). We performed single silencing of HDAC1 and 2 and found there was no significant change with H3K27AC. In contrast with double silencing of HDAC1 and HDAC2 (Additional file 1: Figure S1). These results indicated that H2AZK4/7AC and H3K27AC may have a synergistic effect on the malignant progression of GBM.

### PI3K/AKT pathway blockade released the EGFR-vIII-mediated inhibition of H2AZK4/7AC

To investigate whether activation of the PI3K/AKT pathway participated in the downregulation of H2AZK4/7AC, LY294002 was used to treat U87-vIII, U251-vIII, and N9-vIII cells; we found that H2AZK4/7AC and H3K27AC expression was increased. Conversely, the expression of H3K27me3 was decreased. We found no change in H3K4me3 levels (Fig. 3e).

### Bioinformatics analysis showed that EGFR negatively regulated USP11, SELK, HIP1R, CYFIP2, and ALAD expression

**Fig. 4:**
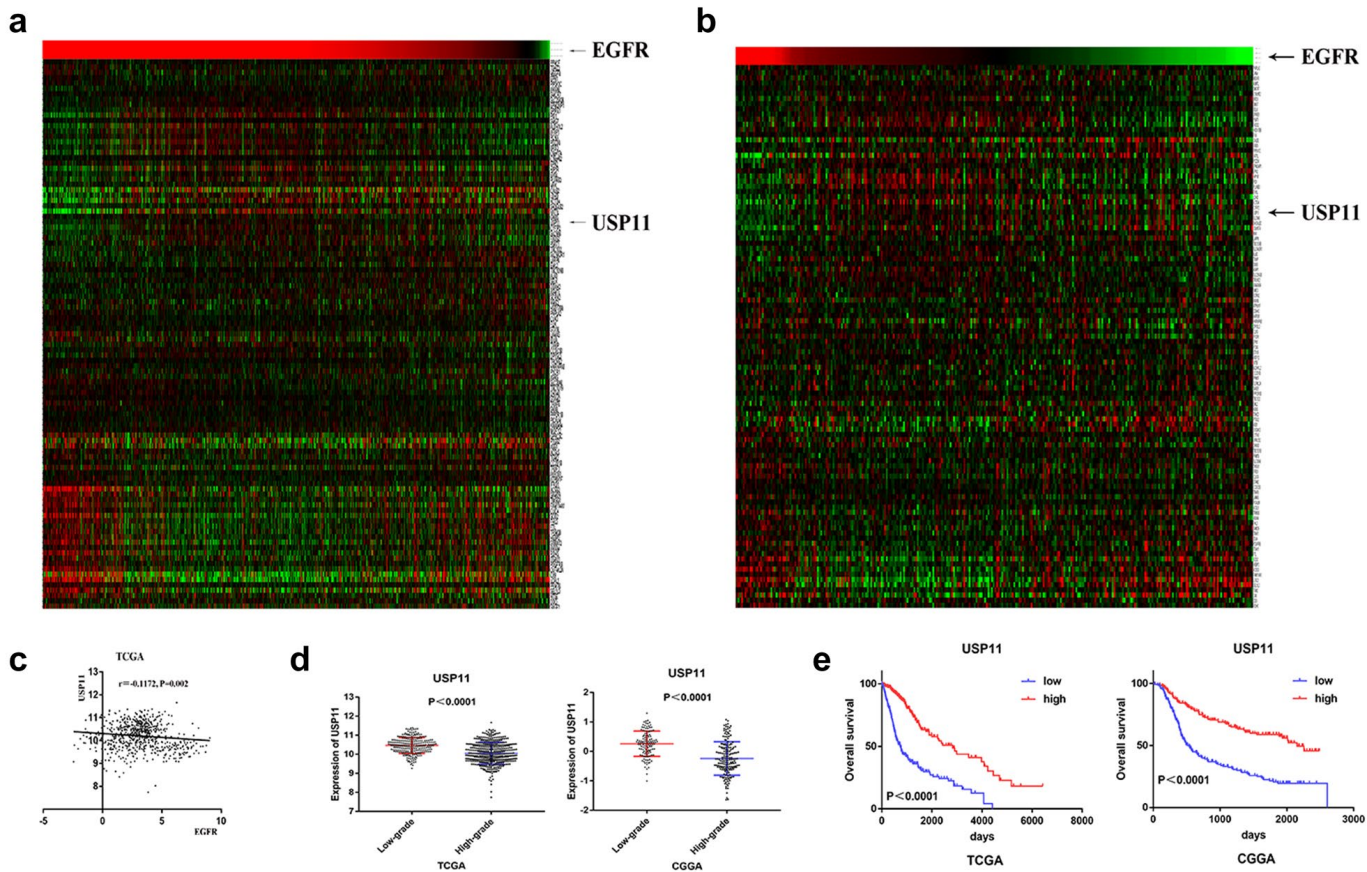
EGFR negatively regulated USP11 expression. **a**, **b** Cluster analysis of data from the CGGA and TCGA databases (106 genes). **c** Pearson correlation analysis showed that USP11 expression was negatively correlated with EGFR. **d** The expression levels of USP11 were negatively correlated with GBM grade. **e** High expression levels of USP11 were associated with a better prognosis in glioma

**Fig. 5:**
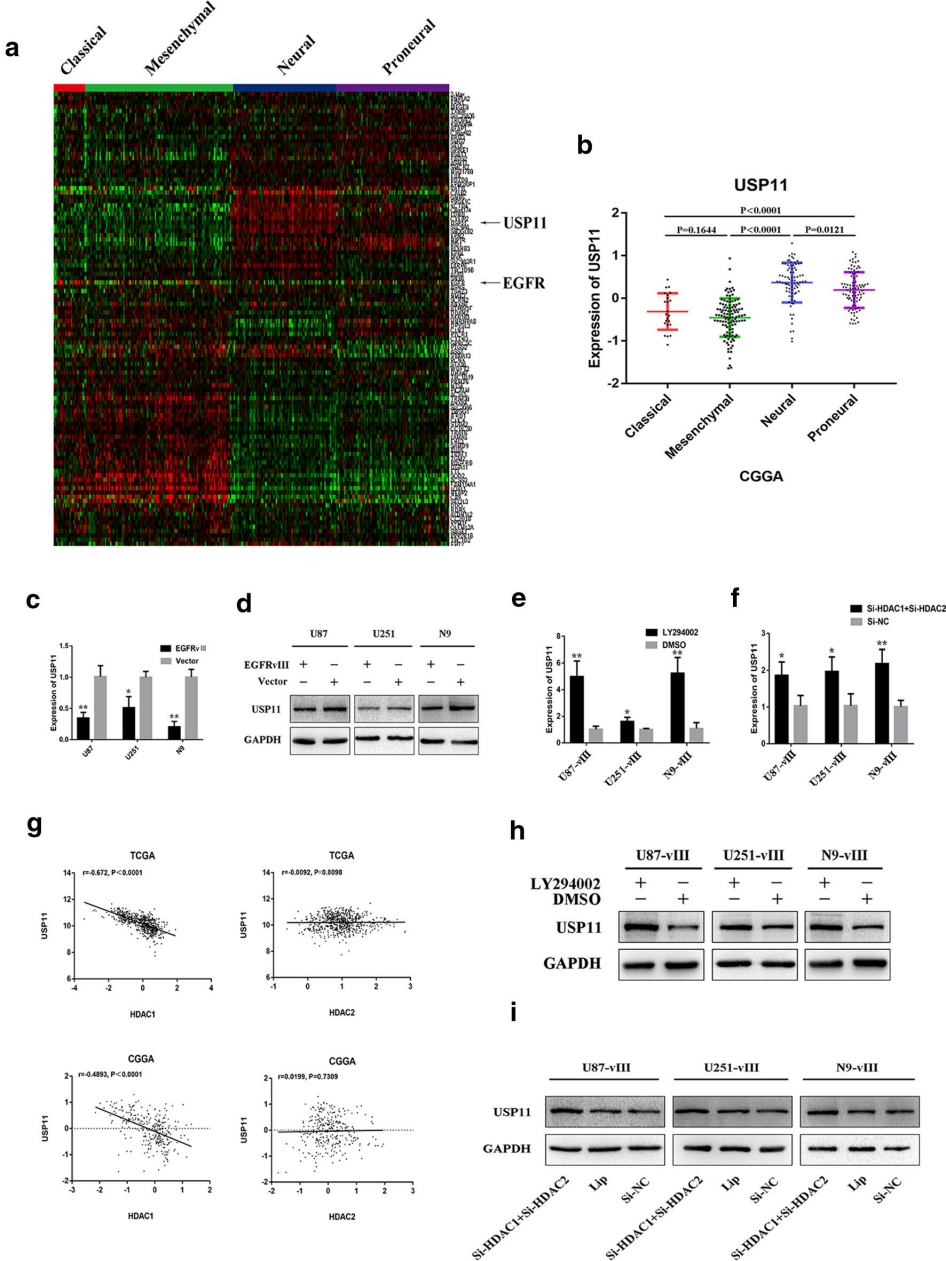
EGFR-vIII enhanced USP11 promoter silencing through the PI3K/AKT-HDAC1/2 axis. **a**, **b** Cluster analysis of data from the CGGA showed that USP11 were expressed mainly in the neural and proneural subtypes of GBM, while EGFR was principally expressed in the classic subtype of GBM. **c**, **d** EGFR-VIII downregulated USP11 at the mRNA and protein levels in U87-vIII, U251-vIII and N9-vIII cells. **e**, **h** PI3K/AKT pathway inhibition upregulated the transcription and translation of USP11 in U87-vIII, U251-vIII and N9-vIII cells. **f**, **i** The knockout of both HDAC1 and HDAC2 upregulated USP11 at the mRNA and protein levels in U87-vIII, U251-vIII and N9-vIII cells

We subjected the total proteins of U87-vIII and U87 cells to mass spectrometry analysis. Data in the CGGA and TCGA public databases were analysed to search for genes negatively regulated by EGFR-vIII. Cluster analysis of data from the CGGA and TCGA databases (106 genes) and the results of Pearson correlation analysis showed that USP11, SELK, HIP1R, CYFIP2 and ALAD expression levels were negatively correlated with EGFR (Fig. 4a–c, Additional file 2: Figure S2A–C). These 106 genes were shown by mass spectrometry analysis to be expressed at lower levels in U87-vIII cells than in U87 cells. Grade-related analysis showed that the expression levels of USP11, SELK, HIP1R, CYFIP2 and ALAD were negatively correlated with grade (Fig. 4d, Additional file 2: Figure S2D) and that high expression levels of USP11, SELK, HIP1R, CYFIP2 and ALAD were significantly associated with a better prognosis (Fig. 4e, Additional file 2: Figure S2E). Cluster analysis of gene expression with data from different subtypes of GBM from the CGGA showed that USP11, SELK, HIP1R, CYFIP2 and ALAD were expressed mainly in the neural and proneural subtypes of GBM, while EGFR was principally expressed in the classic subtype of GBM [12] (Fig. 5a, b, Additional file 3: Figure S3a, b). These results indicated that activation of EGFR/EGFR-vIII might promote the malignant progression of GBM through downregulating USP11, SELK, HIP1R, CYFIP2 and ALAD.

### EGFR**-**vIII activation inhibited USP11 expression through a PI3K/Akt-mediated epigenetic pathway

USP11 is a deubiquitinase that binds several substrates maintains their stability though their deubiquitination [44]. Evidence has indicated that USP11 has anti-tumour activity in lung adenocarcinoma [45] and glioma [46]. To demonstrate that USP11 is downregulated upon EGFR-vIII activation, Western blotting and RT-qPCR were performed and showed that USP11 expression was downregulated at the mRNA and protein levels in U87, U251 and N9 cells upon the activation of EGFR-vIII (Fig. 5c, d). Emerging evidence has shown that the PI3K/AKT pathway can inhibit USP11 transcription and translation. LY294002 was used to treat U87-vIII, U251-vIII and N9-vIII cells, and PI3K/AKT pathway inhibition resulted in the transcriptional and translational upregulation of USP11 (Fig. 5e, h). The USP11 promoter-associated increase in H3 acetylation and decrease in H3K27 trimethylation were reported to result in the transcriptional activation of USP11 [46]. Bioinformatics analysis showed a significant negative correlation between HDAC1 and USP11 (Fig. 5 g). The knockout of both HDAC1 and HDAC2 upregulated USP11 at the mRNA and protein levels in U87-vIII, U251-vIII and N9-vIII cells (Fig. 5f, i).

**Fig. 6:**
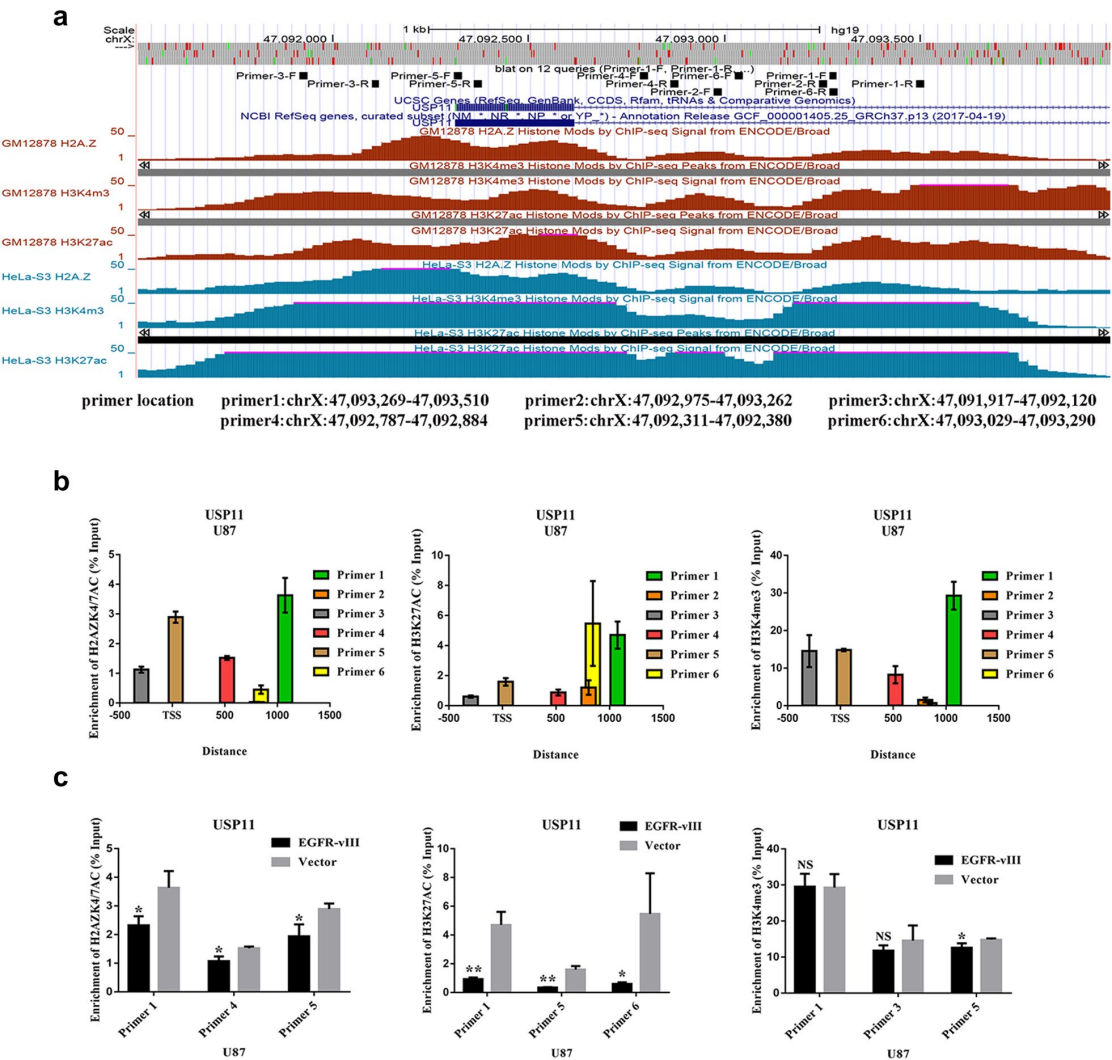
EGFR-vIII enhanced USP11 promoter silencing through downregulating H3K27AC and H2AZK4/7AC in the USP11 promoter area. **a** H3K4me3-, H3K27AC- and H2AZ-binding sites and the locations of six primers. **b** The locations of the predicted primers based on TSS. **c** ChIP-qPCR results showed that the enrichment of H3K27AC and H2AZK4/7AC in the USP11 promoter area was decreased in U87 cells when EGFR-vIII was activated

We have proven that the simultaneous knockout of HDAC1 and HDAC2 upregulated H2AZK4/7AC and H3K27AC. Analysis of data from the UCSC website revealed that the USP11 promoter area was enriched in H3K4me3, H3K27AC and H2AZ (Fig. 6a). Predicted primers (primers 1, 2, 3, 4, 5, and 6) distributed around USP11 transcription start sites (TSSs) are shown in Fig. 6b. The three primers with the highest degree of enrichment for each histone were selected for ChIP-qPCR. The results showed that the USP11 promoter area specifically bound H3K4me3, H3K27AC and H2AZK4/7AC. The enrichment of the USP11 promoter area for H3K27AC and H2AZK4/7AC was decreased in U87 cells upon EGFR-vIII activation (Fig. 6c). We found no change in the enrichment of the USP11 promoter area for H3K4me3 (Fig. 6c). These results demonstrated that H3K27AC and H2AZK4/7AC synergistically enhanced USP11 expression in U87 cells. In addition, EGFR-vIII enhanced silencing of the USP11 promoter through the PI3K/AKT-HDAC1/2 axis.

### FK228 induced G1/S transition arrest in vitro

A dynamic balance between histone acetylation and deacetylation is maintained to regulate gene expression appropriately; a disturbance in this balance in cancer though altered gene expression can accelerate cell cycle progression [47]. FK228, which is a specific HDAC1 and HDAC2 inhibitor, was confirmed to induce cell cycle arrest in the G1/S phase in hepatocellular carcinoma [29]. To investigate whether FK228 can block the cell cycle, we analysed the cell cycle distribution. A decrease in the proportion of cells in S phase and a corresponding increase in the proportion of cells in G1 phase were found in U87-vIII, U251-vIII and N9-vIII cells after treatment with FK228 (Additional file 4: Figure S4A). We also examined the levels of cell cycle-related protein in FK228-treated cells. We found that the levels of cyclin D1, CDK4 and CDK6, all of which were expressed in cells in G1 phase, were reduced in U87-vIII, U251-vIII and N9-vIII cells treated with FK228. In contrast, P21, which negatively regulates the cell cycle, was significantly upregulated after FK228 treatment (Additional file 4: Figure S4B).

### FK228 suppressed GBM xenograft growth in nude mice

**Fig. 7:**
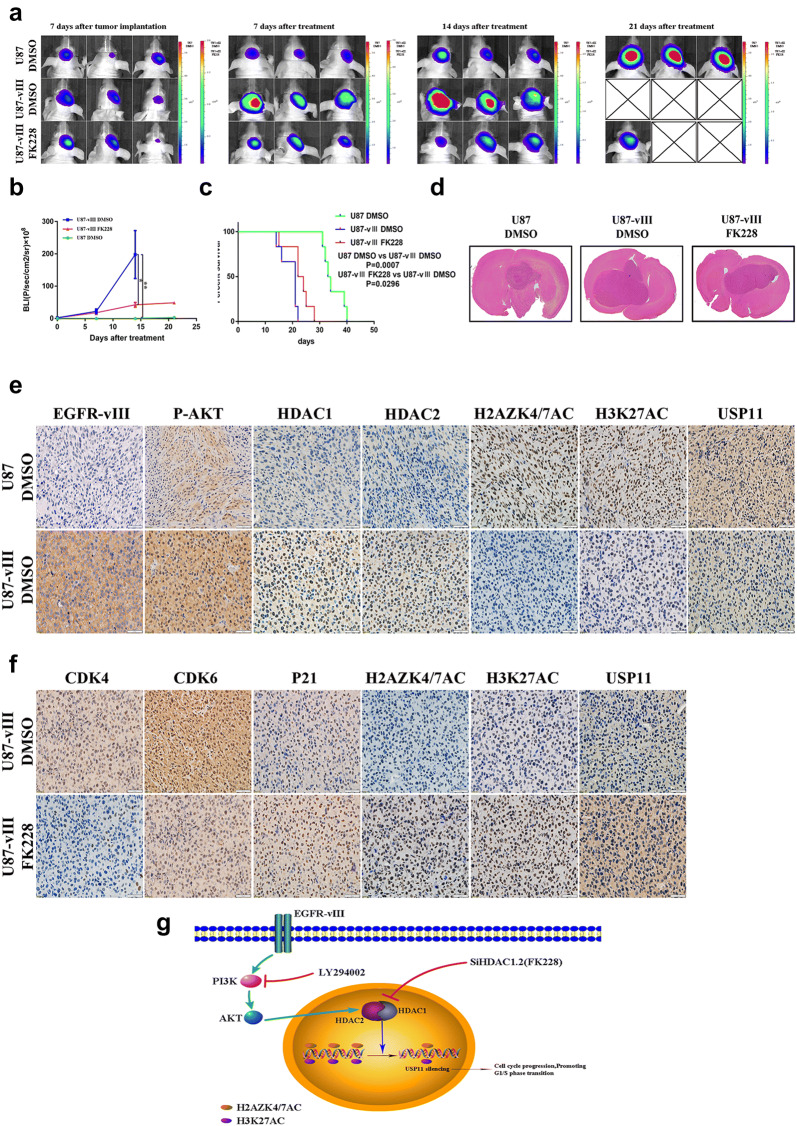
FK228 suppressed GBM cell xenograft growth in nude mice. **a** The extent of tumour growth in DMSO-treated U87 cell xenografts, DMSO-treated U87-vIII cell xenografts and FK228-treated U87-vIII cell xenografts was measured by bioluminescence imaging. **b**, **d** The FK228-treated group showed significantly inhibited tumour growth compared with the DMSO-treated group. **c** Survival analysis among the three groups. **e** EGFR-vIII activation significantly upregulated the expression of P-AKT, HDAC1 and HDAC2 and downregulated H2AZK4/7AC, H3K27AC and USP11 expression. **f** FK228 treatment decreased the expression of CDK4 and CDK6 and increased P21 expression. In addition, FK228 treatment upregulated H2AZK4/7AC and H3K27AC expression and enhanced USP11 expression. **g** The mechanism suggested by the results of the study

We used U87 and U87-vIII cells to construct an in vivo GBM model. To further evaluate the therapeutic efficacy of FK228 in GBM with EGFR-vIII, we treated U87-vIII cell xenograft GBM intracranial model mice with FK228 (1 µg/g) every 3 days. Bioluminescence imaging showed the degree of tumour growth in DMSO-treated U87 cell xenograft mice, DMSO-treated U87-vIII cell xenograft mice and FK228-treated U87-vIII cell xenograft mice (Fig. 7a). The FK228-treated group showed significantly inhibited tumour growth compared with the DMSO-treated groups (Fig. 7b, d). In addition, the FK228-treated mice exhibited significantly longer survival times (Fig. 7c). IHC staining showed that EGFR-vIII activation significantly upregulated the expression of P-AKT, HDAC1 and HDAC2 and downregulated H2AZK4/7AC, H3K27AC and USP11 expression (Fig. 7e). In addition, FK228 treatment decreased the expression of CDK4 and CDK6 and increased P21 expression (Fig. 7f). Furthermore, FK228 treatment upregulated H2AZK4/7AC and H3K27AC expression and enhanced USP11 expression (Fig. 7f). These results indicated that FK228 suppressed U87-vIII cell tumour growth and induced cell cycle arrest in vivo and that EGFR-vIII epigenetically silenced USP11, while FK228 partially released this inhibition.

## Discussion

GBM, the most common brain tumour in adults, has a poor prognosis. Surgical excision in combination with adjuvant radiotherapy and chemotherapy has limited benefits. Therefore, the need to identify the potential mechanisms resulting in GBM malignant progression and develop corresponding effective targets is urgent. The EGFR-vIII mutation is a common malignant event in GBM. Deng and his colleagues found that the deubiquitinase USP11 could inhibit cell cycle progression from G1 to S phase though stabilizing P21 [25]. In our study, we found for the first time that H2AZK4/7AC downregulation contributed to GBM malignant progression when EGFR-vIII was activated. We demonstrated that the EGFR-vIII mutation downregulated H2AZK4/7AC and H3K27AC, inhibiting USP11 expression though the PI3K/AKT-HDAC1/2 axis. FK228, a specific HDAC1 and HDAC2 inhibitor, was shown to have anti-tumour effects though inducing G1/S transition arrest in GBM in vitro and in vivo. Our findings provide persuasive evidence that the histone deacetylase inhibitor FK228 can remodel the cancer epigenome to induce cell cycle progression arrest, affording new insights for the exploration of prospective targets for GBM therapies.

HDAC1 expression was increased at the mRNA and protein levels upon EGFR-vIII activation in U87 and N9 cells but not in U251 cells. This might be the reason for the differences in cancer gene expression between U251 cells and U87 and N9 cells, which are GBM cell lines with different subtypes. The concurrent blockade of HDAC1 and HDAC2 induced cell cycle arrest, but the specific mechanisms of this effect in GBM cells were not clear. In our study, the concurrent inhibition of HDAC1 and HDAC2 upregulated USP11 and induced G1/S transition arrest in GBM cells in vitro and in vivo. USP11 was reported to have tumour-suppressive functions in brain tumours [46] and serve as a negative regulator of the cell cycle [25]. We assume FK228-induced G1/S transition arrest had an anti-tumour effect in GBM though epigenetic remodelling to upregulate USP11 expression. This work will continue in future research.

Our present work characterized the functional interactions between HDAC2 and H2AZK4/7AC. Previous researchers found that H2AZ could be acetylated by Esa1 and Gcn5 in yeast [20, 48], but the mechanism of human histone H2AZ acetylation is still unclear. We demonstrated for the first time that histone H2AZ could be acetylated at lysine residues 4 and 7 by HDAC2 in human cancer cells. This finding provides new insight into the potential role of HDAC2 in tumorigenesis. Since Bonenfant and his colleagues observed the acetylation of human replacement histone H2AZ at lysine residues 4 and 7 for the first time [49], the function of H2AZAC has received extensive attention. H2AZAC was reported to play an important role in active gene expression at promoter regions in cancer, and the deacetylation of H2AZ was found to induce gene repression [50]. However, the specific function of H2AZK4/7AC in GBM is still poorly understood. Our results indicated that H2AZK4/7AC combined with H3K27AC bound promoter regions to enhance USP11 expression in U87 cells. When EGFR-vIII was activated, the binding of H2AZK4/7AC and H3K27AC at USP11 promoter regions was reduced, which led to USP11 inhibition. These findings provide new insight into the role of H2AZK4/7AC in GBM tumorigenesis.

Epigenetic abnormalities have been associated with tumour development and progression and are considered novel therapeutic targets. Several studies have suggested that HDAC inhibitors can inhibit tumour development and progression though inducing cell cycle arrest, inducing apoptosis, reducing chemotherapy resistance and inhibiting angiogenesis [51–53]. FK228 has been used in phase II trials to treat breast cancer (https://clinicaltrials.gov). The EGFR-vIII mutation is the most common malignant event in GBM, and there is no effective treatment for GBM with EGFR-vIII mutation thus far. Therefore, the need to develop a new treatment strategy for EGFR-vIII GBM is urgent. In our study, we demonstrated for the first time that FK228 could induce cell cycle arrest to treat GBM with EGFR-vIII mutation. FK228 could enhance anti-tumour activity against GBM by upregulating the expression of the tumour suppressor USP11. Our results indicate a new therapeutic target and an effective treatment for GBM with EGFR-vIII mutation. Significant progress has been made in the treatment of gliomas with drug combinations [54]. Our study might inspire the combination of FK228 and other small molecule inhibitors with other drugs, although further studies are needed.

## Conclusions

In summary, we found for the first time that H2AZK4/7AC downregulation contributed to GBM malignant progression when EGFR-vIII was activated. EGFR-vIII mutation can downregulate H2AZK4/7AC and H3K27AC, inhibiting USP11 expression though the PI3K/AKT-HDAC1/2 axis. FK228 was shown to have anti-tumour effects though inducing G1/S transition arrest in GBM in vitro and in vivo. Our findings provide persuasive evidence that the histone deacetylase inhibitor FK228 can remodel the cancer epigenome to induce cell cycle progression arrest, affording new insights for the exploration of prospective targets for GBM therapies.

## Supplementary information

**Additional file 1: Figure S1.** Single silencing of HDAC1 and HDAC2 could not up-modulated the expression of H3K27AC. (A) Single silencing of HDAC1 and HDAC2 showed that there was no significant change with H3K27AC in U87-vIII, U251-vIII and N9-vIII cells.

**Additional file 2: Figure S2.** EGFR negatively regulated SELK, HIP1R, CYFIP2, and ALAD expression. (A, B) Cluster analysis of data from the CGGA and TCGA databases (106 genes). (C) Pearson correlation analysis showed that SELK, HIP1R, CYFIP2 and ALAD expression was negatively correlated with EGFR. (D) The expression levels of SELK, HIP1R, CYFIP2 and ALAD were negatively correlated with GBM grade. (E) High expression levels of SELK, HIP1R, CYFIP2 and ALAD were associated with a better prognosis in glioma.

**Additional file 3: Figure S3.** SELK, HIP1R, CYFIP2 and ALAD were expressed mainly in the neural and proneural subtypes. (A, B) Cluster analysis of data from the CGGA showed that SELK, HIP1R, CYFIP2 and ALAD were expressed mainly in the neural and proneural subtypes of GBM, while EGFR was principally expressed in the classic subtype of GBM.

**Additional file 4: Figure S4.** FK228 induced G1/S transition arrest in vitro. (A) FK228 induced G1/S transition arrest in U87-vIII, U251-vIII and N9-vIII cells. (B) FK228 reduced cyclin D1, CDK4, and CDK6 expression and increased P21 expression.

## Data Availability

The datasets analysed during the current study are available in CGGA (http://www.cgga.org.cn/), TCGA (https://xenabrowser.net/datapages/, TCGA.GBMLGG.sampleMap/HiSeqV2) and UCSC (http://genome.ucsc.edu).

## Abbreviations

HATs: histone acetyltransferases
HDACs: histone deacetylases
GBM: glioblastoma
EGFR-vIII: EGFR variant III
CGGA: the Chinese Glioma Genome Atlas
TCGA: the Cancer Genome Atlas
ATCC: American Type Culture Collection
DMEM: Dulbecco’s-modified Eagle medium
FBS: foetal bovine serum
RT-qPCR: real-time quantitative PCR
ChIP: chromatin immunoprecipitation
Co-IP: co-immunoprecipitation
H&E: haematoxylin–eosin
IHC: immunohistochemical
USP11: ubiquitin-specific protease 11
TSS: transcription start site
PI: propidium iodide
