# Supplementary figures and images for: EGFR‐vIII downregulated H2AZK4/7AC though the PI3K/AKT‐HDAC2 axis to regulate cell cycle progression
Source: Clin Transl Med. 2020 Jan 28;9(1):e10. doi: 10.1186/s40169-020-0260-7 (PMC6987283; doi:10.1186/s40169-020-0260-7)

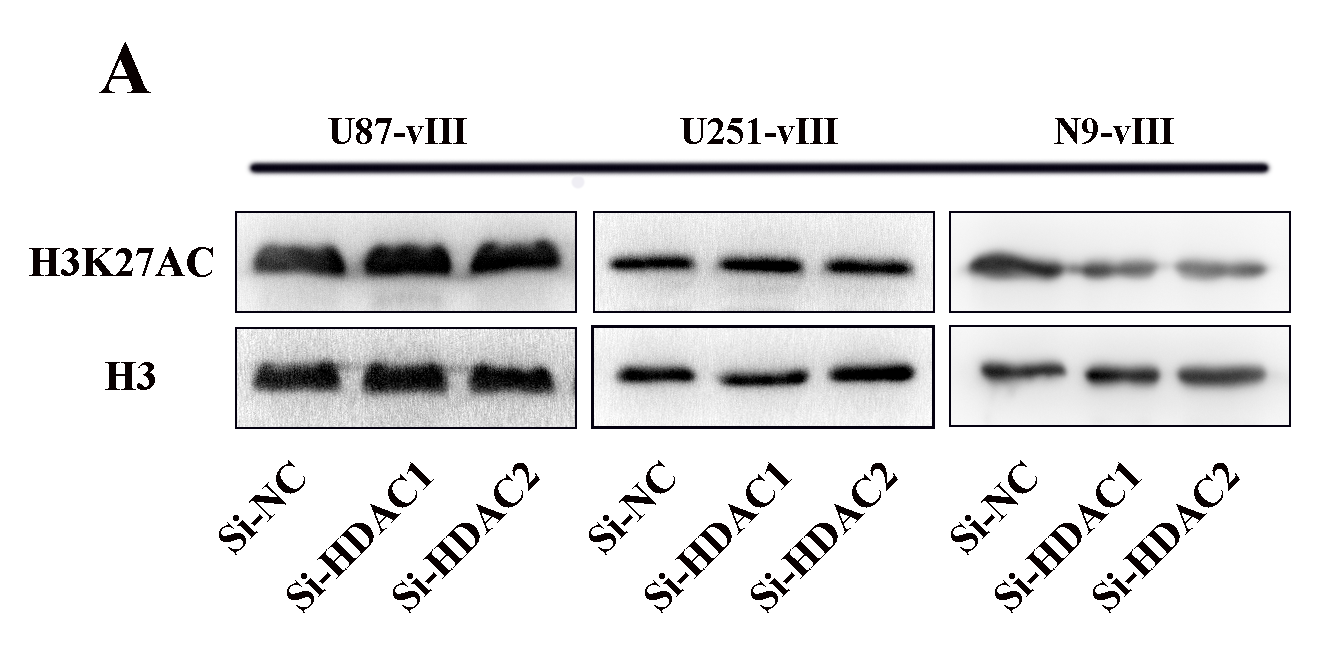

Supplement: Supplementary file 1 — Additional file 1: Figure S1. Single silencing of HDAC1 and HDAC2 could not up‐modulated the expression of H3K27AC. (A) Single silencing of HDAC1 and HDAC2 showed that there was no significant change with H3K27AC in U87‐vIII, U251‐vIII and N9‐vIII cells. [file CTM2-9-0-s001.tif]

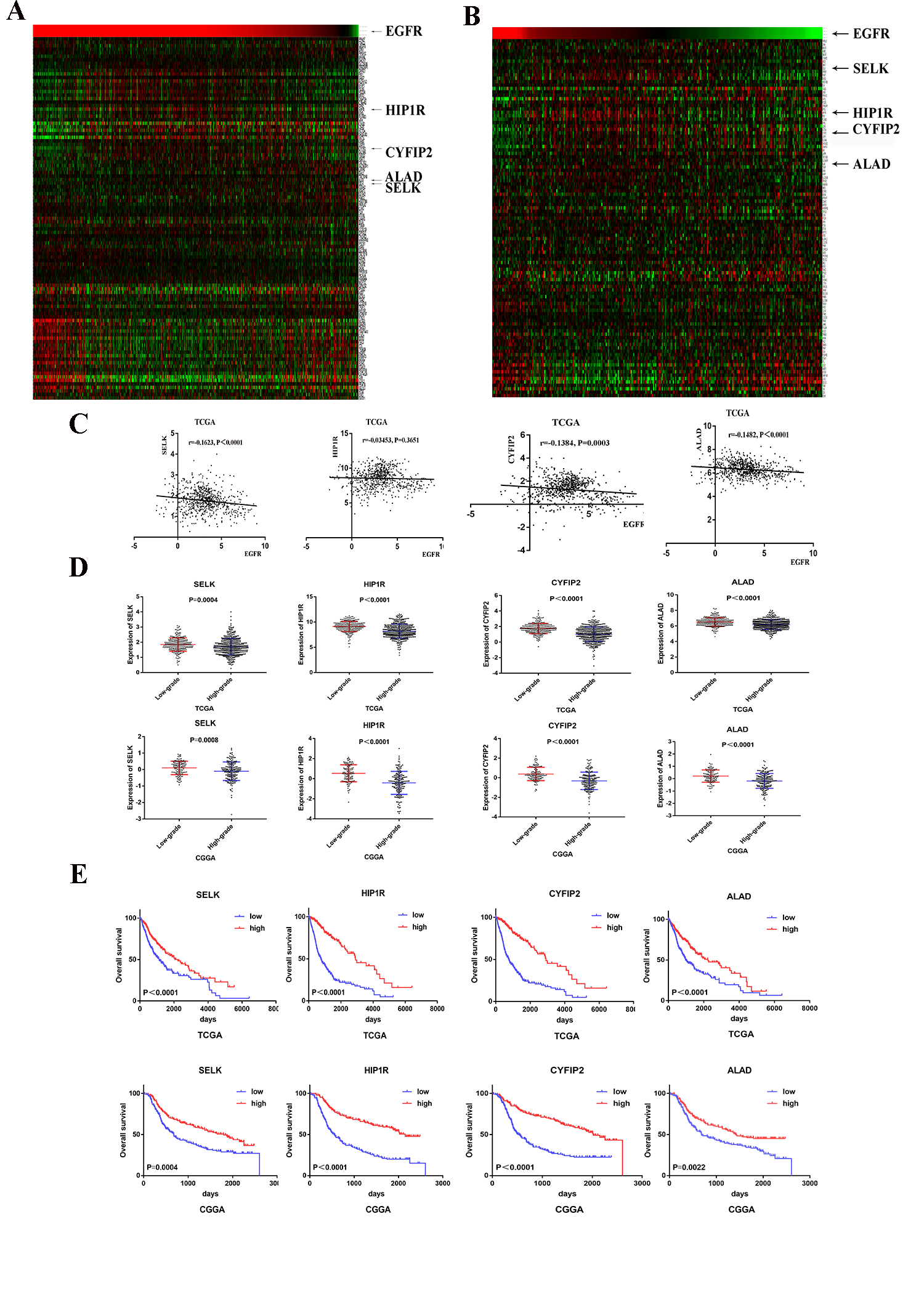

Supplement: Supplementary file 2 — Additional file 2: Figure S2. EGFR negatively regulated SELK, HIP1R, CYFIP2, and ALAD expression. (A, B) Cluster analysis of data from the CGGA and TCGA databases (106 genes). (C) Pearson correlation analysis showed that SELK, HIP1R, CYFIP2 and ALAD expression was negatively correlated with EGFR. (D) The expression levels of SELK, HIP1R, CYFIP2 and ALAD were negatively correlated with GBM grade. (E) High expression levels of SELK, HIP1R, CYFIP2 and ALAD were associated with a better prognosis in glioma. [file CTM2-9-0-s002.tif]

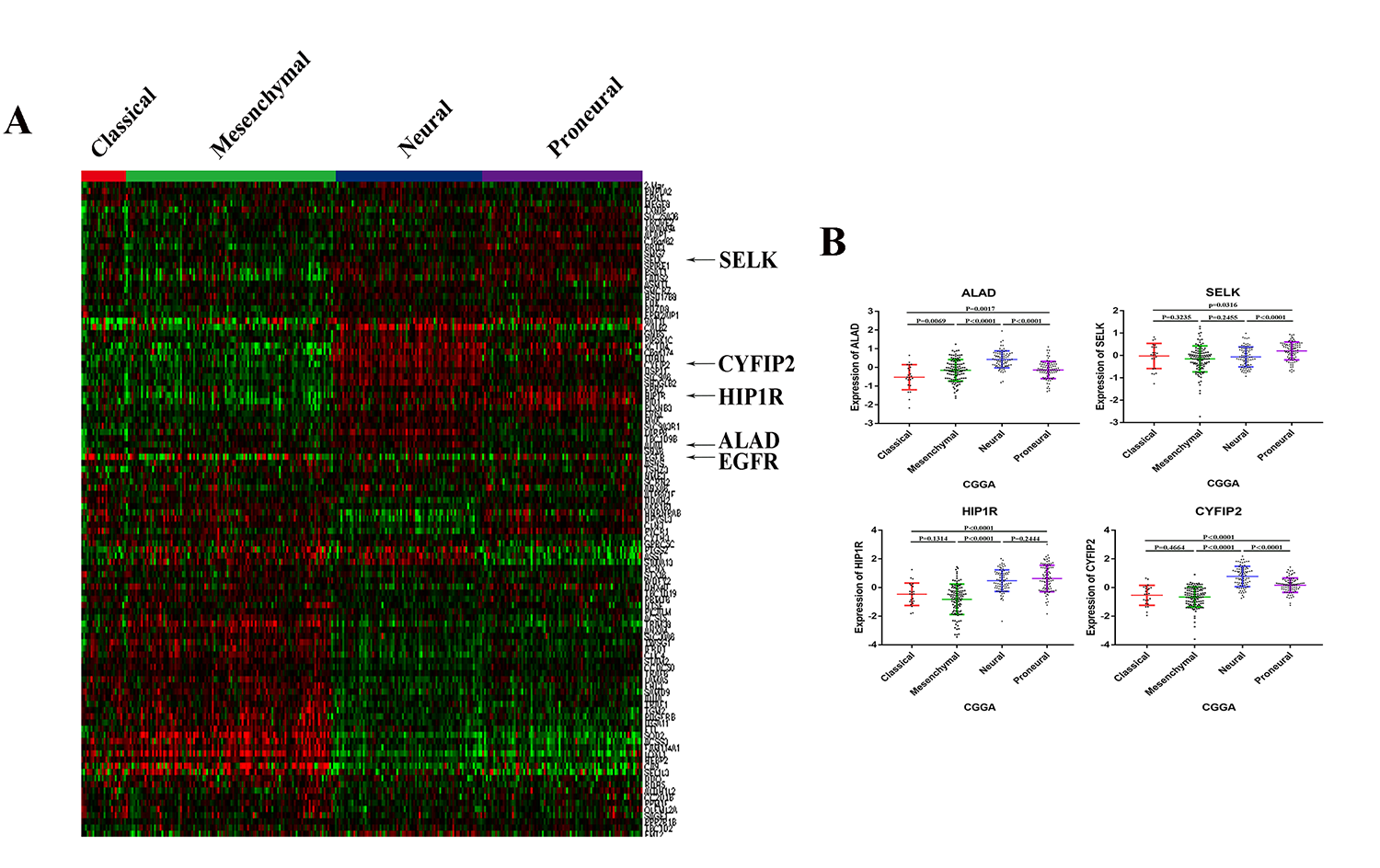

Supplement: Supplementary file 3 — Additional file 3: Figure S3. SELK, HIP1R, CYFIP2 and ALAD were expressed mainly in the neural and proneural subtypes. (A, B) Cluster analysis of data from the CGGA showed that SELK, HIP1R, CYFIP2 and ALAD were expressed mainly in the neural and proneural subtypes of GBM, while EGFR was principally expressed in the classic subtype of GBM. [file CTM2-9-0-s003.tif]

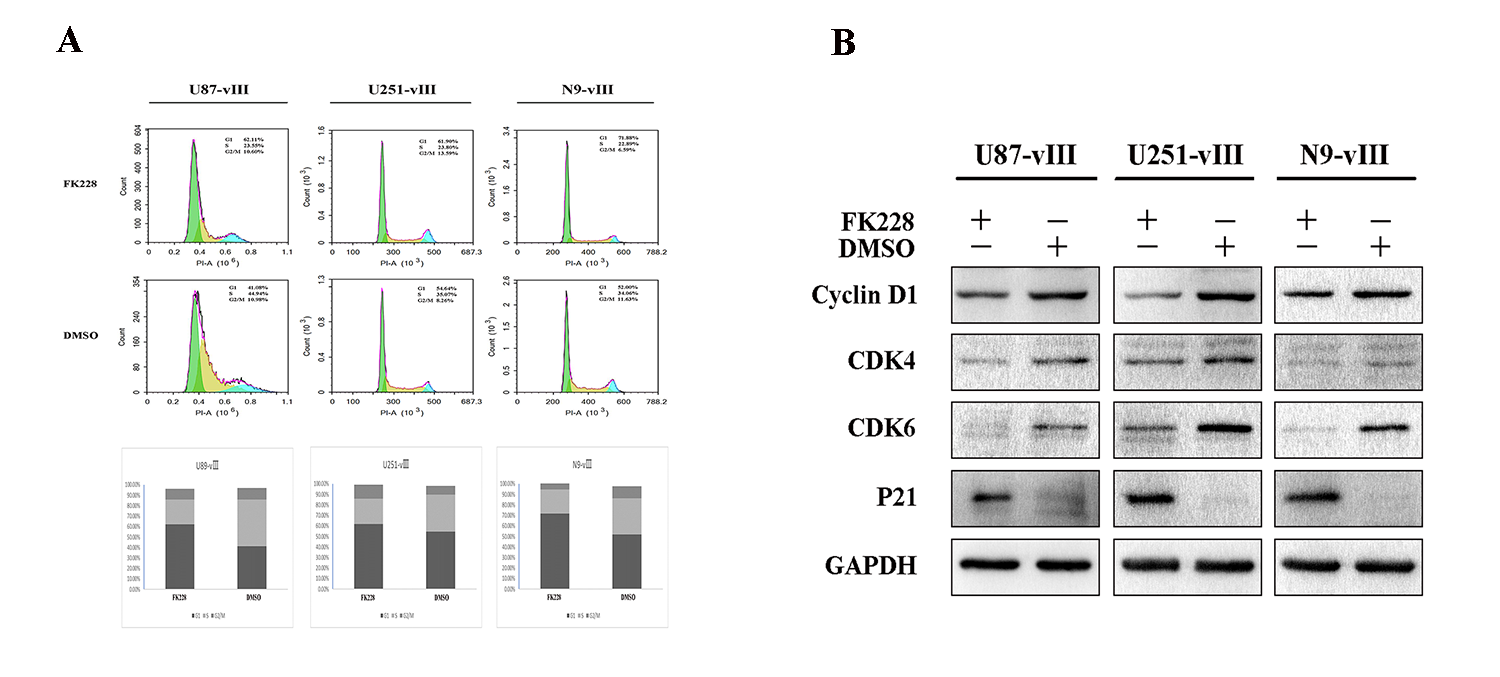

Supplement: Supplementary file 4 — Additional file 4: Figure S4. FK228 induced G1/S transition arrest in vitro. (A) FK228 induced G1/S transition arrest in U87‐vIII, U251‐vIII and N9‐vIII cells. (B) FK228 reduced cyclin D1, CDK4, and CDK6 expression and increased P21 expression. [file CTM2-9-0-s004.tif]
